# Central Dopaminergic System and Its Implications in Stress-Mediated Neurological Disorders and Gastric Ulcers: Short Review

**DOI:** 10.1155/2012/182671

**Published:** 2012-09-13

**Authors:** Naila Rasheed, Abdullah Alghasham

**Affiliations:** ^1^Department of Medical Biochemistry, College of Medicine, Qassim University, P.O. BOX 6655, Buraidah 51452, Saudi Arabia; ^2^Department of Pharmacology and Therapeutics, College of Medicine, Qassim University, P.O. BOX 6655, Buraidah 51452, Saudi Arabia

## Abstract

For decades, it has been suggested that dysfunction of dopaminergic pathways and their associated modulations in dopamine levels play a major role in the pathogenesis of neurological disorders. Dopaminergic system is involved in the stress response, and the neural mechanisms involved in stress are important for current research, but the recent and past data on the stress response by dopaminergic system have received little attention. Therefore, we have discussed these data on the stress response and propose a role for dopamine in coping with stress. In addition, we have also discussed gastric stress ulcers and their correlation with dopaminergic system. Furthermore, we have also highlighted some of the glucocorticoids and dopamine-mediated neurological disorders. Our literature survey suggests that dopaminergic system has received little attention in both clinical and preclinical research on stress, but the current research on this issue will surely identify a better understanding of stressful events and will give better ideas for further efficient antistress treatments.

## 1. Introduction

Dopamine (DA) is an important endogenous catecholamine, which exerts widespread effects on both neuronal (as a neurotransmitter) and nonneuronal tissues (as an autocrine or paracrine agent) [1]. Within the central nervous system (CNS), DA binds to specific membrane receptors presented by neurons, and it plays a key role in the control of locomotion, learning, working memory, cognition, and emotion [2, 3]. The brain DA system is involved in various neurological and psychiatric disturbances including Parkinson's disease, schizophrenia, amphetamine, and cocaine addiction [1, 3]. Therefore, it is considered to be a major target for drug designing applied in the treatment of neurological diseases. Stress has been shown to alter normal dopaminergic neurotransmission [4], and exposure to stress profoundly increases the dopaminergic activity [4, 5] and induces relevant adaptive response of DA receptors in specific brain regions [6]. Stress also activates the hypothalamus-pituitary-adrenal (HPA) axis and releases glucocorticoids (GCs). The interplay between GCs and the dopaminergic system is linked with various neurological disorders such as schizophrenia, bipolar depressive disorder and major depressive disorder, addiction, and Parkinson's disease [7, 8]. A number of reports showed the involvement of GCs on DA-mediated behavioral responsiveness by the modulatory effects of corticosterone [8–10]. Many reports suggest the involvement of DA system in locomotors alterations under different stressful conditions [9–11]. The stress-induced adaptation of brain DA function involves receptors, and it has also been demonstrated that DA receptor densities are affected by altered extracellular DA levels [10, 12, 13]. It is also demonstrated that stress manipulations induced the alteration in motor activity of experimental animals, and dopaminergic pathways are crucial to neural substrates for the control of spontaneous locomotor activity [3, 11]. These studies clearly indicated that DA plays an essential role in locomotion via neural transmission. 

Dopaminergic system is also known to play a regulatory role in gastric ulcers under various stressful conditions [14, 15]. Patients with Parkinson's disease have higher rate of ulcer, where DA becomes deficient. But in patients with schizophrenia, DA level usually becomes high, and the rate of gastric ulcer becomes very less [15]. This indicates that DA levels must have a link with gastric pathology. Studies also suggested that modulation of dopaminergic transmission induced by DA drugs facilitates the gastric cytomodulatory effects [15, 16]. Furthermore, administration of DA or related agents attenuated stress ulcerogenesis, whereas opposite effects have been also seen with DA-lytic drugs [15–17]. Not only this, but it is also reported that DA mediates gastric cytoprotective effects on other neurotransmitters [18, 19]. Now, it is well established that stress induced ulcerogenesis is governed by activation of the mesocorticolimbic DA systems [15–20]. Alterations of DA levels and total ulcer score in acute and chronic unpredictable stress models have been summarized in Table 1. All this debate clearly indicates that DA plays an important role in ulcerogenesis during stress. This paper provides an update on DA activities in stressful events that represent, in our opinion, the optimal utility as future therapeutic target for neurodegenerative disorders. 

## 2. Interplay between Stressful Events and Central Dopaminergic System

In 1950, Hns Selye borrowed the term “stress” from physics and hypothesized that a constellation of stereotypic psychological and physiological events occurring in seriously ill patients represented the consequences of a severe, prolonged application of adaptation responses. He recognized that stress plays a very significant role in the development of all types of diseases [21]. Selye believed that daily lives are influenced by two different kinds of stress: pleasant stress contributing to “wellness” and unpleasant stress contributing to disease and sickness [21]. Mesocortical and mesolimbic (M-L) dopaminergic systems are known to mediate HPA axis-induced GC release and other CNS effects [8, 22, 23]. Various neurological disorders are linked with GCs and the dopaminergic system [7, 8, 23]. Evidence shows that central dopaminergic system exerts positive effects on the HPA axis and the sympathetic nervous system (SNS), and reciprocally, glucocorticoids and catecholamines mediated stress-induced alterations [24, 25]. Modulations of DA in major brain regions are mediated by dopaminergic receptors, which are classified as D_1_ and D_2_ types. Classification of these DA receptors is based on the mechanism that links these G-protein-coupled receptors (GPCRs) to the second messenger system [26]. Thus, D_1_-like receptors stimulate the adenylate cyclase activity via Gs subunit leading to an increased cyclic adenosine monophosphate (cAMP) concentration [27]. On the other hand, D_2_-like receptors are negatively coupled via the Gi subunit to the adenylate cyclase, which leads to a decline in the cAMP concentration. Both D_1_ and D_2_ of receptors are abundantly expressed in major brain areas such as nucleus accumbens, striatum, frontal cortex, amygdala, and hippocampus [27]. Furthermore, both D_1_ and D_2_ are also involved in vigilance, hormonal homeostasis, and locomotor activities. It is reported that stressful experiences alter DA metabolism through D_1_ and D_2_ receptors and release in the M-L system [28–30]. Furthermore, it is also reported that exposure to a single unavoidable/uncontrollable aversive experience may lead to inhibition of DA release in the nucleus accumbens as well as to impair the response to both rewarding and aversive stimuli [25, 31]. The effects of stressful experiences on DA functioning in the M-L system can be very different or even opposite depending on situation, the genetic background of the organism, and its life history [24]. We and the others have shown that stress differentially increases the dynamics of DA depending on the brain regions involved [9, 30]. Reports also stated that stressful stimuli tend to cause the largest increase in DA levels in the PFC (prefrontal cortex) region, with markedly smaller changes in the limbic and dorsal striatal regions [32, 33]; however, this relationship is altered by lesions of different nuclei. Thus, stress causes release of DA in the amygdala, and lesions of the amygdala tend to block stress-induced increases in PFC DA levels [34]. Lesions of the PFC also affect this response. Studies in which the PFC DA innervations are lesioned show that subsequent stressors cause a much larger increase in DA levels within the nucleus accumbens, particularly with respect to the duration of the response [31, 34]. This suggested that PFC DA released in response to stress actually blunts the responsiveness of the subcortical limbic DA system. In contrast, 6-OHDA lesions of PFC DA levels were found to decrease the basal electrophysiologic activity of ventral tegmental area (VTA) DA neurons [35]. Repeated stress also has important clinical implications in regard to the DA system. A recent study examined how chronic stress in the form of cold exposure affects the discharge of VTA DA neurons. Thus, after exposing rats to cold, there was a 64% decrease in the number of spontaneously active DA neurons, with no significant alteration in their average firing rate. Nonetheless, there was a subpopulation of neurons that exhibited excessive burst activity in the exposed rats [36]. Unlike acute exposure to stressful or noxious stimuli, chronic stress actually attenuates DA neuron baseline activity. 

The interplay between glucocorticoids (GCs) and the dopaminergic systems has been reported in many human diseases [37]. GCs are released as a result of HPA-axis activation in stressful condition [7, 8, 38]. Mesocortical and M-L dopaminergic neuronal systems are hypothesized to mediate some of the CNS effects of glucocorticoids [7, 22, 38]. Our previous study favors this hypothesis, in which we found elevated levels of corticosterone and alteration in GCs receptor in different brain regions during stress [39, 40]. The fact that both corticosterone and DA are sensitive to both psychological and physical environmental stimuli suggests that the interaction between these two chemical messengers may be involved in mediating the differential responding to positively reinforcing drugs following a single or repeated stressful experience. This is further supported by various other investigators that provide evidence for a decreased prefrontal dopaminergic transmission. Adrenalectomy impaired working memory resulted in decreased dopaminergic transmission in the PFC [41]. Furthermore, addition of GCs can increase dopaminergic activity in PFC, suggesting a crosstalk between the GCs receptor and the dopaminergic system. Taken all together, these data suggest that, both GCs and DA systems represent attractive therapeutic targets for stress-induced neurological disorders and should be investigated further. Modulations of dopaminergic pathways and their associated changes of dopamine levels in neurological disorders have been shown in Table 2.

Here, we have discussed some of the DA- and GCs-mediated neurological diseases.

### 2.1. Schizophrenia

Millions of people suffer from schizophrenia at some point in their life, making it one of the most common health problems in the whole world [8]. This biological disorder of the brain is a result of abnormalities, which arise early in life and disrupt the normal development of the brain. These abnormalities involve structural differences between a schizophrenic brain and a healthy brain [14]. The role of HPA axis changes in patients with schizophrenia is currently a matter of debate. Now, it is well established that hyperactivity of HPA axis is one of the parts for pathogenesis of schizophrenia. First, reduced GR gene expression levels, studied mainly by *in situ* hybridization assays, have been described in the frontal cortex and throughout all the hippocampus subfields of schizophrenic patients [8, 52, 53]. Second, neuropathological brain changes observed in schizophrenia are similar as changes caused by increased GC levels [54]. Conclusions should be made with caution as quantitative mRNA versus protein expression studies do not always result in a GR signal change of the same magnitude. Furthermore, it is also possible that these findings may be a downstream effect of the primary etiology or could be epiphenomena or even the effect of a drug treatment. One of the negative symptoms of schizophrenia is an impairment of working memory (the short-term storage needed for certain tasks). Several research groups have reported that HPA disruption leads to working memory impairment [55–57]. Furthermore, addition of GCs can increase dopaminergic activity in PFC, which suggests a crosstalk between the GR and the dopaminergic system [41]. 

Schizophrenic brains under stressful conditions tend to have larger lateral ventricles and a smaller volume of tissue in the left temporal lobe in comparison to healthy brains [58], and the chemical nature of a schizophrenic brain is different in the manner the brain handles DA in stressful (GC secretion) events [8]. Thousands of chemical processes take place in a functioning neuron. The transfer of information is mediated by neurotransmitters that interact with certain receptors [8]. A study was conducted in which presynaptic DA function (measured by the uptake of fluorodopa) was observed by positron emission tomography (PET) in the brains of seven schizophrenic patients and eight healthy people (controls). The fluorodopa influx constant was higher in the schizophrenic patients. Their receptors took up more fluorodopa [58]. In conclusion, these alterations in presynaptic DA function during stressful conditions constituted a part of the disrupted neural circuits that predispose people to schizophrenia [58–60]. The DA receptors involved in these processes can be separated into the D_1_ and D_2_ families. The D_1_ family contains the receptors D_1_ and D_5_. The D_1_ receptors in the brain are linked to episodic memory, emotion, and cognition. These functions are disturbed in schizophrenic patients during stressful conditions. In addition, D_1_ binding of DA was found to be lower in schizophrenic patients as compared to healthy subjects of the same age. The binding was lower as a result of fewer D_1_ receptors. Certain antipsychotic drugs stimulate D_1_-regulated pathways, which increases the D_1_ to D_2_ activity balance in the brain. This balance can also be regained by the release of DA. Not much is known about D_5_ due to the lack of drugs that are selective for it. The D_2_ family contains the receptors D_2_, D_3_, and D_4_. D_2_ is the second most abundant DA receptor in the brain. D_2_ receptor blockade is the main target for antipsychotic drugs, because there is a higher density of D_2_ in schizophrenic brains under stressful conditions [8, 58–60]. Studies have shown a selective loss of D_3_ mRNA expression in the parietal and motor cortices of postmortem, schizophrenic brains [61]. This phenomenon may be due to either the course of the disease or therapy given to the patients. Studies have also found that the density of D_4_ receptors was elevated sixfold in schizophrenic patients. These DA receptors are affected by alterations in the neural cell membranes, which could disrupt communication between cells. Abnormalities in two long-chain fatty acids in the blood cells of people with negative symptoms have been discovered. These substances break down into products that are involved in the DA system [59]. DA is secreted by cells in the midbrain that send their axons to the basal ganglia and frontal lobe. Certain drugs used for schizophrenia bind to the DA receptors. This blocks DA binding to the receptor. This deactivates the biochemical processes normally initiated by DA binding. First, DA binds to the receptor, and then the receptor autophosphorylates. By phosphorylation, this receptor activates adenylate cyclase, which then makes cAMP. These processes involve the synthesis of cAMP and synaptic action at synapses using DA as a transmitter. The DA synapses are incapacitated by antipsychotic drugs. DA antagonists are drugs that block DA receptors. The brain responds to this receptor blockade by making extra DA receptors. This is the postsynaptic cells' attempt to compensate for the weakening of synaptic transmission, which is caused by the drugs. These extra receptors restore the cell's sensitivity to DA. The brain also compensates by increasing DA synthesis. The increase in DA synthesis lasts one to two weeks of medication from the start of therapy, which is the same time required for the medication to become effective. Drugs have been discovered to alleviate the upregulation of receptors and the increased synthesis of DA [62]. Antischizophrenic drugs are called neuroleptics. A DA antagonist is chlorpromazine (Thorazine), and reserpine operates by depleting transmitter stores. Ligand-binding techniques, which use neuroleptic drugs labeled with radioisotopes, demonstrate that such drugs bind to DA receptors. A correlation exists between this ability to bind DA and the dosage required to improve schizophrenic symptoms in patients. This effect could also be directly observed by PET in living subjects [58]. Controlling DA and DA receptors is essential for the treatment of schizophrenia. Because schizophrenia is hereditary, it is important to see progress for the next generation [59]. In the future, there will be more sophisticated drugs that do not merely suppress symptoms but also allow for normal cognitive functioning. Although schizophrenics or stressful events may never be normal, they can be made tolerable.

### 2.2. Parkinson's Disease

Parkinson's disease (PD) is a devastating neurodegenerative disorder affecting several million people worldwide. It inflicts a tremendous social and economic burden on modern society where the incidence of the disease increases with age [8]. Currently, the mean age of onset is around 55 years. In all cases, the clinical features which characterize PD, including resting tremor, bradykinesia, and postural instability, are progressive [63]. Distinct among the pathological features of PD is the significant loss of dopaminergic neurons in the substantia nigra leading to a dramatic depletion of DA in the striatum. Although neurological disorders are present in every population and PD is one of them, treatment of PD is still limited to a few drugs such as levodopa. The etiology of PD is still not completely understood, but neuroinflammation is an important contributor to the neuronal loss in the disease [64]. Indeed, few drugs have been reported to partially inhibit microglial reaction, to decrease the production of proinflammatory cytokines and NO, and thus to attenuate the degeneration of DA-containing neurons in *in vivo* PD models [8, 65, 66]. While in humans these drugs provide relief from symptoms, however, none of them has been shown to inhibit disease progress; they also have varying degrees of side effects [67]. Therefore, there is an urgent need for novel neuroprotective agents for the treatment of PD patients. 

It is not obvious if an immediate pathological link exists between the dopaminergic and the GCs systems in this disorder. Affecting these systems can relieve some of the symptoms of Parkinson's disease, for example, raising the DA levels in patients improves their working memory deficit [68, 69]. Evidence for an interaction between GR and DA pathways in the region of the brain, involved in PD, comes from studies with transgenic mice, expressing less GR [70]. These mice show increased concentrations of DA, DA D_1_, and D_2_ receptor ligand binding in the striatum and decreased binding to dopamine transporter in the substantia nigra resulting in a sensitization of dopaminergic functions [70]. The foregoing discussion indicates that it is not clear to what extent the pathological link exists between the GCs and DA systems in PD and its utility as monotherapy in this disorder, but data clearly suggests their roles in PD and supports further studies. 

### 2.3. Bipolar Depressive and Major Depressive Disorders

Depressive disorders present another example of a connection between stress axis dysregulations and a psychiatric illness [70–73]. It has been reported that in psychotic major depression (PMD), the psychotic symptoms may be due to an increase in DA activity and synthesis secondary to HPA axis over activity [8, 74]. Numerous reports suggest interactions between the HPA axis and the central dopaminergic system contributing to the development of delusions and cognitive deficits in psychotic major depression [55, 75]. In experiments with depressed and schizophrenic patients, assessing the effect of DA receptor agonists on multiple hormone levels, some investigators [76] could not find a causal link between HPA axis hyperactivity and DA dysregulation to explain psychotic symptoms in psychotic major depression. However, other symptoms of depression, such as impaired cognitive functions, can be related with DA neurotransmission [77]. Several antidepressants are also reported to enhance DA transmission and improve working memory impairment in patients [78], suggesting a link between HPA axis and DA in PMD. In addition, the use of mifepristone such as RU486, the morning-after pill, a GC antagonist which primarily blocks GRs in the PFC of the brain, has been reported to ameliorate psychosis and depression in patients with Cushing's disease [79, 80] and even turned out to be quickly effective to treat PMD in cases of little responsiveness to combination therapies of antipsychotics and antidepressants [81, 82]. These results strongly suggest that the psychosis observed in PMD is caused by HPA axis over activation. Some mood stabilizers are also reported to inhibit the transcriptional activity of GR and thus inhibit the detrimental effect of excess GCs on the central nervous system [83]. Reciprocally, transgenic mice overexpressing GR specifically in the forebrain display a significant increase in anxiety-like and depressive behaviors. They are also supersensitive to antidepressants and show enhanced sensitization to cocaine. This phenotype is associated in specific brain regions with increased expression of genes relevant to emotionality [84]. 

In view of these data, this indicates a crosstalk with the dopaminergic system and supports the general hypothesis that GC hormonal disturbances can indeed lead to the development of disorders. Furthermore, it indicates that natural variations in GR gene expression can contribute to the fine tuning of emotional stability or liability and play a role in bipolar disorder and may represent an attractive therapeutic target in patients with these disorders.

### 2.4. Addiction

Stress is known to facilitate the psychostimulant self-administration, which represents an indication for the degree of addiction. Adrenalectomized animals studies have shown a consistently lower drug intake as compared to control animals. Subsequent administration of corticosterone up to hormonal stress levels resulted in a restoration of DA receptor agonist responses in a dose-dependent manner. Importantly, the effect of GC (stress) abolishment on self-administration cannot be attributed to nonspecific decreases in motivation or motor behavior, respectively, as seeking behavior for food is not affected [85]. It is also reported that adrenalectomy reduces the extracellular concentrations of DA in the shell of the accumbens (Acb), both basally and after psychostimulant administration, providing evidence for an interaction between GCs and DA [86, 87]. These effects were most probably GR dependent, because GR antagonists also induced a drop in DA Acb shell levels, whereas the usage of MR antagonists had no effect [88]. Deletion of GR in the nervous system, using the Cre-loxP recombination system, also results in a loss of sensitization after cocaine treatment, confirming the important role for GR signaling in DA-related emotional behavior [89]. GC-activated GR thus enhances drug responding by selectively facilitating dopaminergic transmission in the shell of the Acb. Studies monitoring DA levels after stress-induced GC secretion, exogenous GC administration, or in a background of high endogenous GC levels are more controversial. For example, the group of Chrousos found that chronic hypercortisolemia rather inhibits dopamine synthesis and turnover in the Acb [90]. It is clear, however, by using the same tools (adrenalectomy or pharmacological blockade of GC production) that GCs are implicated in stress-induced sensitization to psychostimulants as well as in the relapse to drug-seeking behavior induced by stress [91]. Of importance, the key for developing stress-induced sensitization is possibly a long-term exposure to high levels of corticosterone as opposed to an acute treatment.

**Figure 1:**
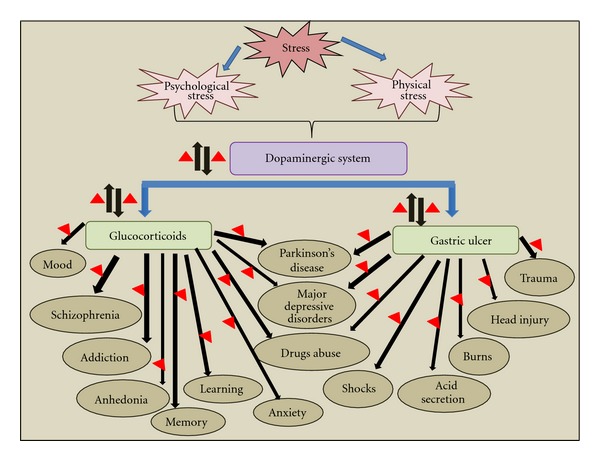
Overview of stress-induced dopaminergic modulations and their associated changes in glucocorticoid and gastric ulcer. Stressful stimuli lead to dopamine release in the brains of animals or humans. The number of neurological disorders has been linked to the dopaminergic modulated response due to physiological or psychological stressors via perturbations in glucocorticoids and gastric ulcer. Up and down arrows together indicate modulations, and triangles indicate possible therapeutic targets.

Stress is a contributing factor, and DA is a fundamental regulator of neurological diseases including substance use disorders, anxiety, depression, and schizophrenia. Therefore, DA or its receptors should be therapeutic targets for controlling the stress and for prevention of the onset of stress-related neurological disorders. Now, it is well established that GCs and DA have an important role in maintaining normal brain functions and the molecular and mechanistic aspects of GC effects on normal functioning of brain and behavior with the specific reference to DA signaling. Therefore, GCs, DA and DA signaling are emerging therapeutic targets for interdisciplinary research field that addresses the interplay between neuronal and endocrine signaling in psychiatric disorders.  Figure 1 summarizes an overview on stress-induced modulations in dopaminergic system and its associated pathological conditions. In addition, possible therapeutic targets have also been mentioned.

## 3. Dopamine and Gastric Stress Ulcers

**Figure 2:**
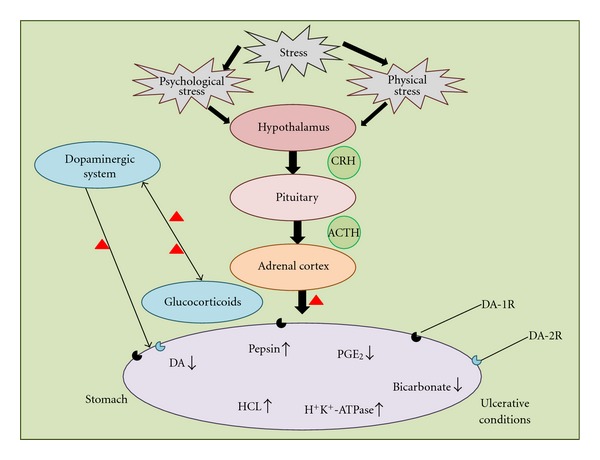
Stress-induced modulations in dopaminergic system and gastric ulcer. Hormonal pathways by which psychological and physical stress induce modulations in stomach functioning, resulting in an increase production of gastric ulceration and modulation of dopaminergic system. Up arrows indicate increased response, down arrows indicate decreased response, triangles indicate possible therapeutic targets. Abbreviations: CRH: corticotrophin-releasing hormone; ACTH: adrenocorticotrophin-releasing factor; PGE_2_ and prostaglandin E_2_; HCL: hydrochloric acid; H^+^K^+^-ATPase: hydrogen-potassium ATPase: DA: dopamine; DA-1R: DA receptor 1; DA-2R: DA receptor 2.

Among the various neurotransmitters, the dopaminergic system, in particular, plays an important regulatory role in stress-induced gastric ulcers [6–10]. Interestingly, in DA deficiency diseases (such as Parkinson's disease), the degree of ulceration was found to be higher [92, 93]; whereas in patients having DA excess amount (such as Schizophrenia), the degree of ulceration was found to be lower [92, 93], this clearly indicate a link between DA levels and gastric pathology. The modulation in dopaminergic transmission by specific DA drugs is also known to affect on gastric cytomodulatory functions [94]. Other contributing factors of DA system to stress ulcers are increased gastric motility, vagal overactivity, decreased gastric mucosal blood flow, and various other neuroendocrinological factors [95–97]. Elevated corticosteroid level is also known to modulate gastric glands to secrete acid and pepsin, which further deteriorate gastric mucosal integrity [97–100]. Stress-mediated peptic ulcer has been involved in various neuropathological conditions [97]. Brain-gut axis plays an important role in controlling gastric functions for various brain neurochemical factors during stress ulcer disease [15]. As early as 1965, Strang [101] noted an apparent association between central DA and peripheral gastric disease in those Parkinson's disease patients, characterized by central DA deficiency, exhibited a higher-than-expected incidence of ulcer disease. Later, Szabo [102] confirmed a protective role for DA in an experimental model of duodenal ulcer. Now, connection between DA activity and gastroduodenal ulcer disease is well established [18, 103]. A number of pharmacological agents have now been designed and tested that showed protective role against brain dysfunctioning [104, 105], but whether they have antiulcer activity that remains to be investigated other than our paper [39]. Previously, we have shown that a drug A68930 has antistress activity in acute and chronic unpredictable stress models [39]. In the same paper, we have shown that stimulated dopaminergic receptors (D_1_/D_2_) modulate the activity gastric H^+^K^+^-ATPase and PGE_2_ levels in acute and chronic unpredictable stress models, and the stress-induced gastric ulceration could be attributed to the stimulation of paraventricular nucleus of hypothalamus, increased intestinal motility, acid secretion, and so forth [39, 106, 107]. This has been summarized in Figure 2.

 Elevated corticosteroid levels are known to modulate gastric glands to secrete acid and pepsin [108], which can further deteriorate gastric mucosal integrity. It is well known that the gastric tissue is under reciprocal control of cholinergic (stimulatory) and adrenergic (inhibitory) autonomic fibers, and an intimate connection exists between the sympathoadrenal system and mucosal integrity, suggesting that the decrease in gastric dopamine levels during stress may be associated with the disruption of normal tone of sympathetic and parasympathetic actions. Gastric cytomodulatory effects are also proposed through the modulation of dopaminergic transmission by specific DA drugs. For example, both central and peripheral administration of DA and related agents attenuated stress ulcerogenesis, whereas opposite effects were observed with DA-lytic drugs [16–19]. DA is also reported to mediate gastric cytoprotective effects of other neurotransmitters [18, 19]. In 1981, Willems et al. [109] suggested that there exist two distinct DA receptor subtypes in the periphery (DA_1_ and DA_2_). Glavin [110] tested several of these compounds for their ability to influence restraint stress ulcerogenesis. The selective DA_1_ agonist SKF38393,markedly reduced restraint stress-induced ulcers as well as ethanol-induced gastric lesions and basal gastric acid secretion. The selective DA_1_ antagonist SCH23390 worsened stress ulcers, ethanol ulcers, and augmented gastric secretion. DA_2_ selective compounds (N-0434, N-0437, quinpirole, eticlopride) were inactive against stress ulcer formation. Additional support for mesolimbic DA as a critical site in mediating gastrointestinal responses to stress challenge comes from Kauffman's group [111], who showed that neurotensin-induced protection against stress ulcerogenesis requires intact mesolimbic DA for the full expression of this effect DA antagonist administered into terminal fields of the mesolimbic DA tract significantly obtund the antiulcer activity of neurotensin. These results, together with those of Henke, strongly implicate central DA, and in particular mesolimbic DA acting through D_1_ receptors, as an important endogenous gastroprotective system [112]. There exists a significant role for DA as an endogenous protective element against stress-related gastroduodenal mucosal injury. Both central and peripheral DA contributes to this effect, likely through D_1_/DA_1_ receptors. It also appears likely that mesolimbic DA, preferentially activated by stress challenge, is primary mediator of central component of DA-induced gastroprotection. 

Data revealed herein may gather importance in respect of several facts. The results provide insights into the role of dopaminergic system in modulating various aspects of stress and gastric pathology through the stimulation of specific dopamine receptors. Gastroprotective effects of antistress drugs may have clinical relevance, as stress-induced gastric injury and bleeding are the major causes for death of patients suffering from shock, trauma, and massive burns [113, 114].

## 4. Conclusion

Despite the power of modern molecular or pharmacological approaches and persisting investigative efforts, the complete interaction between the mesocorticolimbic dopaminergic system and stress activation remains to be identified. Recent advancements have contributed to the recognition of dopaminergic innervation as a useful system for determining reactions to perturbations in environmental conditions, for selective information processing and for controlling emotional behavior, all of which play an essential role in the ability (or failure) to cope with the external world. Now, it is well established that stressful events provoke major behavioral, neurochemical, and gastric ulcerative effects involving mesocorticolimbic DA functioning, but the type of alterations induced by these experiences remains highly controversial, but it may depend on the behavioural situation and genetic makeup of the organism. Exposure to uncontrollable aversive experiences leads to inhibition of DA release in the mesoaccumbens DA system as well as impaired responding to rewarding and aversive stimuli. Repeated and chronic stressful experiences can reduce the capability of stressors to disrupt behavior, induce behavioral sensitization to psychostimulants, and to promote adaptive changes of mesolimbic DA functioning. For the last two decades, studies aimed to develop new pharmacological approaches to search for drugs devoid of behaviorally sensitizing effects and capable of protecting the organism against the devastating effects of adaptation to stress. This paper updates the current knowledge on the physiological regulation of DA neurons by glucocorticoids, and gastric ulcer suggests that the blockade of these conditions surely opens new therapeutic strategies for the treatment of neurological disorders.

## Figures and Tables

**Table 1:** Acute and chronic unpredictable stress-induced alterations in dopamine, prostaglandin E_2_ levels, histopathological changes, and mean ulcer score in gastric tissues.

Parameters in gastric tissues	Models		
	Nonstress	Acute stress	Chronic unpredictable stress
Dopamine levels	0	↓	↓↓
Prostaglandin E_2_	0	0	↓
Histopathological changes	0	↑	↑↑
Mean ulcer score	0	↑	↑↑
Plasma corticosterone	0	↑↑	↑

This information was obtained from our previous paper [39]. Plasma corticosterone was shown as stress marker. Symbols represent the following: 0: no effect, ↓: small decrease, ↓↓: large decrease, ↑: small increase, and ↑↑: large increase.

**Table 2:** Modulation of dopaminergic pathways and their associated changes of dopamine levels in neurological disorders.

DA pathways	DA alterations	Disorders	References
Nigrostriatal	DA decrease	Parkinson's disease	[42–45]
		Huntington's disease	[43, 44]
		ADHD	[46]
	DA increase	Schizophrenia	[43]
		Tourette's syndrome	[47]
		ADHD	[43]
Mesocortical	DA increase	Schizophrenia	[43]
		Tourette's syndrome	[43]
Mesolimbic	DA decrease	Epilepsy	[48, 49]
		Drug addiction	[43, 50]
	DA increase	Obesity	[43, 50]
		Depression	[50]
Tuberoinfundibular	DA decrease	Pituitary tumors	[51]

There are four major dopaminergic pathways: (1) nigrostriatal pathway, in which substantia nigra neurons innervate the stratum; (2) nesocortical pathway, which links the ventral tegmental area to medial prefrontal, cingulate, and entorhinal cortices; (3) nesolimbic pathway, composed of ventral tegmental area cells projecting to the nucleus accumbens and other limbic areas; (4) tuberoinfundibular, which projects from arcuate and periventricular nuclei of the hypothalamus to the pituitary gland. Abbreviations: DA: dopamine; ADHD: attention-deficit hyperactivity disorder.

## References

[1] Sarkar C, Basu B, Chakroborty D, Dasgupta PS, Basu S (2010). The immunoregulatory role of dopamine: an update. *Brain, Behavior, and Immunity*.

[2] Nieoullon A, Coquerel A (2003). Dopamine: a key regulator to adapt action, emotion, motivation and cognition. *Current Opinion in Neurology*.

[3] Benturquia N, Courtin C, Noble F, Marie-Claire C (2008). Involvement of D1 dopamine receptor in MDMA-induced locomotor activity and striatal gene expression in mice. *Brain Research*.

[4] Pani L, Porcella A, Gessa GL (2000). The role of stress in the pathophysiology of the dopaminergic system. *Molecular Psychiatry*.

[5] Varga J, Domokos A, Barna I, Jankord R, Bagdy G, Zelena D (2011). Lack of vasopressin does not prevent the behavioural and endocrine changes induced by chronic unpredictable stress. *Brain Research Bulletin*.

[6] Brunelin J, d’Amato T, van Os J, Cochet A, Suaud-Chagny MF, Saoud M (2008). Effects of acute metabolic stress on the dopaminergic and pituitary-adrenal axis activity in patients with schizophrenia, their unaffected siblings and controls. *Schizophrenia Research*.

[7] Daftary SS, Panksepp J, Dong Y, Saal DB (2009). Stress-induced, glucocorticoid-dependent strengthening of glutamatergic synaptic transmission in midbrain dopamine neurons. *Neuroscience Letters*.

[8] Craenenbroeck KV, Bosscher KD, Berghe WV, Vanhoenacker P, Haegeman G (2006). Role of glucocorticoids in dopamine-related neuropsychiatric disorders. *Molecular and Cellular Endocrinology*.

[9] Cabib S, Puglisi-Allegra S (2012). The mesoaccumbens dopamine in coping with stress. *Neuroscience and Biobehavioral Reviews*.

[10] Rasheed N, Ahmad A, Pandey CP, Chaturvedi RK, Lohani M, Palit G (2010). Differential response of central dopaminergic system in acute and chronic unpredictable stress models in rats. *Neurochemical Research*.

[11] Pohorecky LA, Sweeny A, Buckendahl P (2011). Differential sensitivity to amphetamine’s effect on open field behavior of psychosocially stressed male rats. *Psychopharmacology*.

[12] Salamone JD, Correa M, Mingote SM, Weber SM (2005). Beyond the reward hypothesis: alternative functions of nucleus accumbens dopamine. *Current Opinion in Pharmacology*.

[13] Floresco SB (2007). Dopaminergic regulation of limbic-striatal interplay: 2006 CCNP Young Investigator Award. *Journal of Psychiatry and Neuroscience*.

[14] Lodge DJ, Grace AA (2011). Developmental pathology, dopamine, stress and schizophrenia. *International Journal of Developmental Neuroscience*.

[15] Ozdemir V, Jamal MM, Osapay K (2007). Cosegregation of gastrointestinal ulcers and schizophrenia in a large national inpatient discharge database: revisiting the “brain-gut axis” hypothesis in ulcer pathogenesis. *Journal of Investigative Medicine*.

[16] Brzozowski T, Konturek PC, Konturek SJ (2004). Exogenous and endogenous ghrelin in gastroprotection against stress-induced gastric damage. *Regulatory Peptides*.

[17] Landeira-Fernandez J, Grijalva CV (2004). Participation of the substantia nigra dopaminergic neurons in the occurrence of gastric mucosal erosions. *Physiology and Behavior*.

[18] Nishikawa K, Amagase K, Takeuchi K (2007). Effect of dopamine on the healing of acetic acid-induced gastric ulcers in rats. *Inflammopharmacology*.

[19] Saad SF, Agha AM, Amrin AENS (2001). Effect of bromazepam on stress-induced gastric ulcer in rats and its relation to brain neurotransmitters. *Pharmacological Research*.

[20] Degen SB, Geven EJW, Sluyter F, Hof MWP, van der Elst MCJ, Cools AR (2003). Apomorphine-susceptible and apomorphine-unsusceptible Wistar rats differ in their recovery from stress-induced ulcers. *Life Sciences*.

[21] Selye H (1950). Stress and the general adaptation syndrome. *British Medical Journal*.

[22] Radley JJ, Gosselink KL, Sawchenko PE (2009). A discrete GABAergic relay mediates medial prefrontal cortical inhibition of the neuroendocrine stress response. *Journal of Neuroscience*.

[23] Asanuma M, Miyazaki I, Ogawa N (2003). Dopamine- or L-DOPA-induced neurotoxicity: the role of dopamine quinone formation and tyrosinase in a model of Parkinson’s disease. *Neurotoxicity Research*.

[24] Steketee JD, Kalivas PW (2011). Drug wanting: behavioral sensitization and relapse to drug-seeking behavior. *Pharmacological Reviews*.

[25] Cabib S, Puglisi-Allegra S (1996). Stress, depression and the mesolimbic dopamine system. *Psychopharmacology*.

[26] Jaber M, Robinson SW, Missale C, Caron MG (1996). Dopamine receptors and brain function. *Neuropharmacology*.

[27] Missale C, Nash SR, Robinson SW, Jaber M, Caron MG (1998). Dopamine receptors: from structure to function. *Physiological Reviews*.

[28] Imperato A, Angelucci L, Casolini P, Zocchi A, Puglisi-Allegra S (1992). Repeated stressful experiences differently affect limbic dopamine release during and following stress. *Brain Research*.

[29] Belda X, Armario A (2009). Dopamine D1 and D2 dopamine receptors regulate immobilization stress-induced activation of the hypothalamus-pituitary-adrenal axis. *Psychopharmacology*.

[30] Jahng JW, Ryu V, Yoo SB, Noh SJ, Kim JY, Lee JH (2010). Mesolimbic dopaminergic activity responding to acute stress is blunted in adolescent rats that experienced neonatal maternal separation. *Neuroscience*.

[31] Broom SL, Yamamoto BK (2005). Effects of subchronic methamphetamine exposure on basal dopamine and stress-induced dopamine release in the nucleus accumbens shell of rats. *Psychopharmacology*.

[32] Radley JJ, Sawchenko PE (2011). A common substrate for prefrontal and hippocampal inhibition of the neuroendocrine stress response. *Journal of Neuroscience*.

[33] Uehara T, Sumiyoshi T, Matsuoka T, Itoh H, Kurachi M (2007). Effect of prefrontal cortex inactivation on behavioral and neurochemical abnormalities in rats with excitotoxic lesions of the entorhinal cortex. *Synapse*.

[34] Stuber GD, Sparta DR, Stamatakis AM (2011). Excitatory transmission from the amygdala to nucleus accumbens facilitates reward seeking. *Nature*.

[35] Harden DG, King D, Finlay JM, Grace AA (1998). Depletion of dopamine in the prefrontal cortex decreases the basal electrophysiological activity of mesolimbic dopamine neurons. *Brain Research*.

[36] Moore H, Rose HJ, Grace AA (2001). Chronic cold stress reduces the spontaneous activity of ventral tegmental dopamine neurons. *Neuropsychopharmacology*.

[37] Groeneweg FL, Karst H, de Kloet ER, Joëls M (2011). Rapid non-genomic effects of corticosteroids and their role in the central stress response. *Journal of Endocrinology*.

[38] Tafet GE, Bernardini R (2003). Psychoneuroendocrinological links between chronic stress and depression. *Progress in Neuro-Psychopharmacology and Biological Psychiatry*.

[39] Rasheed N, Ahmad A, Singh N (2010). Differential response of A 68930 and sulpiride in stress-induced gastric ulcers in rats. *European Journal of Pharmacology*.

[40] Rasheed N, Ahmad A, Al Sheeha M, Alghasham A, Palit G (2011). Neuroprotective and anti-stress effect of A 68930 in acute and chronic unpredictable stress model in rats. *Neuroscience Letters*.

[41] Mizoguchi K, Ishige A, Takeda S, Aburada M, Tabira T (2004). Endogenous glucocorticoids are essential for maintaining prefrontal cortical cognitive function. *Journal of Neuroscience*.

[42] Gandhi S, Vaarmann A, Yao Z, Duchen MR, Wood NW, Abramov AY (2012). Dopamine induced neurodegeneration in a PINK1 model of Parkinson’s disease. *PLoS ONE*.

[43] Bozzi Y, Borrelli E (2006). Dopamine in neurotoxicity and neuroprotection: what do D2 receptors have to do with it?. *Trends in Neurosciences*.

[44] Bédard C, Wallman MJ, Pourcher E, Gould PV, Parent A, Parent M (2011). Serotonin and dopamine striatal innervation in Parkinson’s disease and Huntington’s chorea. *Parkinsonism and Related Disorders*.

[45] Shen LH, Liao MH, Tseng YC (2012). Recent advances in imaging of dopaminergic neurons for evaluation of neuropsychiatric disorders. *Journal of Biomedicine and Biotechnology*.

[46] Madras BK, Miller GM, Fischman AJ (2005). The dopamine transporter and attention-deficit/hyperactivity disorder. *Biological Psychiatry*.

[47] Singer HS (2005). Tourette’s syndrome: from behaviour to biology. *The Lancet Neurology*.

[48] O’Neill MJ, Hicks CA, Ward MA (1998). Dopamine D2 receptor agonists protect against ischaemia-induced hippocampal neurodegeneration in global cerebral ischaemia. *European Journal of Pharmacology*.

[49] Bozzi Y, Vallone D, Borrelli E (2000). Neuroprotective role of dopamine against hippocampal cell death. *Journal of Neuroscience*.

[50] Park SK, Nguyen MD, Fischer A (2005). Par-4 links dopamine signaling and depression. *Cell*.

[51] Iaccarino C, Samad TA, Mathis C (2002). Control of lactotrop proliferation by dopamine: essential role of signalling through D2 receptors and ERKs. *Proceeding of the National Academy of Sciences USA*.

[52] Webster MJ, Knable MB, O’Grady J, Orthmann J, Weickert CS (2002). Regional specificity of brain glucocorticoid receptor mRNA alterations in subjects with schizophrenia and mood disorders. *Molecular Psychiatry*.

[53] Perlman WR, Webster MJ, Kleinman JE, Weickert CS (2004). Reduced glucocorticoid and estrogen receptor alpha messenger ribonucleic acid levels in the amygdala of patients with major mental illness. *Biological Psychiatry*.

[54] Cotter D, Pariante CM (2002). Stress and the progression of the developmental hypothesis of schizophrenia. *British Journal of Psychiatry*.

[55] Newcomer JW, Selke G, Melson AK (1999). Decreased memory performance in healthy humans induced by stress-level cortisol treatment. *Archives of General Psychiatry*.

[56] Heffelfinger AK, Newcomer JW (2001). Glucocorticoid effects on memory function over the human life span. *Development and Psychopathology*.

[57] Roozendaal B, de Quervain DJF (2005). Glucocorticoid therapy and memory function: lessons learned from basic research. *Neurology*.

[58] Sedvall G, Farde L (1995). Chemical brain anatomy in schizophrenia. *The Lancet*.

[59] Brown P (1994). Understanding the inner voices. *New Scientist*.

[60] Hietala J, Syvalahti E, Vuorio K (1995). Presynaptic dopamine function in striatum of neuroleptic-naive schizophrenic patients. *The Lancet*.

[61] Schmauss C, Haroutunian V, Davis KL, Davidson M (1993). Selective loss of dopamine D3-type receptor mRNA expression in parietal and motor cortices of patients with chronic schizophrenia. *Proceedings of the National Academy of Sciences of the United States of America*.

[62] Lickey M, Gordon B (1990). *Medicine and Mental Illness*.

[63] Fahn S, Sulzer D (2004). Neurodegeneration and neuroprotection in Parkinson disease. *Neurotherapeutics*.

[64] Gao HM, Liu B, Zhang W, Hong JS (2003). Novel anti-inflammatory therapy for Parkinson’s disease. *Trends in Pharmacological Sciences*.

[65] Kurkowska-Jastrzȩbska I, Litwin T, Joniec I (2004). Dexamethasone protects against dopaminergic neurons damage in a mouse model of Parkinson’s disease. *International Immunopharmacology*.

[66] Castaño A, Herrera AJ, Cano J, Machado A (2002). The degenerative effect of a single intranigral injection of LPS on the dopaminergic system is prevented by dexamethasone, and not mimicked by rh-TNF-*α* IL-1*β* IFN-*γ*. *Journal of Neurochemistry*.

[67] Kanthasamy A, Jin H, Mehrotra S, Mishra R, Kanthasamy A, Rana A (2010). Novel cell death signaling pathways in neurotoxicity models of dopaminergic degeneration: relevance to oxidative stress and neuroinflammation in Parkinson’s disease. *NeuroToxicology*.

[68] Lange KW, Robbins TW, Marsden CD, James M, Owen AM, Paul GM (1992). L-Dopa withdrawal in Parkinson’s disease selectively impairs cognitive performance in tests sensitive to frontal lobe dysfunction. *Psychopharmacology*.

[69] Lange KW, Paul GM, Naumann M, Gsell W (1995). Dopaminergic effects on cognitive performance in patients with Parkinson’s disease. *Journal of Neural Transmission, Supplement*.

[70] Cyr M, Morissette M, Barden N, Beaulieu S, Rochford J, Di Paolo T (2001). Dopaminergic activity in transgenic mice underexpressing glucocorticoid receptors: effect of antidepressants. *Neuroscience*.

[71] Caamaño CA, Morano MI, Akil H (2001). Corticosteroid receptors: a dynamic interplay between protein folding and homeostatic control. Possible implications in psychiatric disorders. *Psychopharmacology Bulletin*.

[72] Mizoguchi K, Yuzurihara M, Nagata M, Ishige A, Sasaki H, Tabira T (2002). Dopamine-receptor stimulation in the prefrontal cortex ameliorates stress-induced rotarod impairment. *Pharmacology Biochemistry and Behavior*.

[73] Pariante CM, Miller AH (2001). Glucocorticoid receptors in major depression: relevance to pathophysiology and treatment. *Biological Psychiatry*.

[74] Fleming SK, Blasey C, Schatzberg AF (2004). Neuropsychological correlates of psychotic features in major depressive disorders: a review and meta-analysis. *Journal of Psychiatric Research*.

[75] Lyons DM, Lopez JM, Yang C, Schatzberg AF (2000). Stress-level cortisol treatment impairs inhibitory control of behavior in monkeys. *Journal of Neuroscience*.

[76] Duval F, Mokrani MC, Crocq MA (2000). Dopaminergic function and the cortisol response to dexamethasone in psychotic depression. *Progress in Neuro-Psychopharmacology and Biological Psychiatry*.

[77] de Quervain DJF, Roozendaal B, McGaugh JL (1998). Stress and glucocorticoids impair retrieval of long-term spatial memory. *Nature*.

[78] Calabrese JR, Markovitz PJ (1991). Treatment of depression: new pharmacologic approaches. *Primary Care*.

[79] van der Lely AJ, Foeken K, van der Mast RC, Lamberts SWJ (1991). Rapid reversal of acute psychosis in the Cushing syndrome with the cortisol-receptor antagonist mifepristone (RU 486). *Annals of Internal Medicine*.

[80] Sartor O, Cutler GB (1996). Mifepristone: treatment of Cushing’s syndrome. *Clinical Obstetrics and Gynecology*.

[81] Belanoff JK, Rothschild AJ, Cassidy F (2002). An open label trial of C-1073 (mifepristone) for psychotic major depression. *Biological Psychiatry*.

[82] Chu JW, Matthias DF, Belanoff J, Schatzberg A, Hoffman AR, Feldman D (2001). Successful long-term treatment of refractory Cushing’s disease with high-dose mifepristone (RU 486). *Journal of Clinical Endocrinology and Metabolism*.

[83] Basta-Kaim A, Budziszewska B, Jaworska-Feil L (2004). Mood stabilizers inhibit glucocorticoid receptor function in LMCAT cells. *European Journal of Pharmacology*.

[84] Wei Q, Lu XY, Liu L (2004). Glucocorticoid receptor overexpression in forebrain: a mouse model of increased emotional lability. *Proceedings of the National Academy of Sciences of the United States of America*.

[85] Piazza PV, Le Moal M (1998). The role of stress in drug self-administration. *Trends in Pharmacological Sciences*.

[86] Piazza PV, Barrot M, Rougé-Pont F (1996). Suppression of glucocorticoid secretion and antipsychotic drugs have similar effects on the mesolimbic dopaminergic transmission. *Proceedings of the National Academy of Sciences of the United States of America*.

[87] Piazza PV, Deroche-Gamonent V, Rouge-Pont F, Le Moal M (2000). Vertical shifts in self-administration dose-response functions predict a drug-vulnerable phenotype predisposed to addiction. *Journal of Neuroscience*.

[88] Marinelli M, Aouizerate B, Barrot M, Le Moal M, Piazza PV (1998). Dopamine-dependent responses to morphine depend on glucocorticoid receptors. *Proceedings of the National Academy of Sciences of the United States of America*.

[89] Deroche-Gamonet V, Sillaber I, Aouizerate B (2003). The glucocorticoid receptor as a potential target to reduce cocaine abuse. *Journal of Neuroscience*.

[90] Pacak K, Tjurmina O, Palkovits M (2002). Chronic hypercortisolemia inhibits dopamine synthesis and turnover in the nucleus accumbens: an in vivo microdialysis study. *Neuroendocrinology*.

[91] de Jong IEM, de Kloet ER (2004). Glucocorticoids and vulnerability to psychostimulant drugs: toward substrate and mechanism. *Annals of the New York Academy of Sciences*.

[92] Taché Y, Yang H, Miampamba M, Martinez V, Yuan PQ (2006). Role of brainstem TRH/TRH-R1 receptors in the vagal gastric cholinergic response to various stimuli including sham-feeding. *Autonomic Neuroscience: Basic and Clinical*.

[93] Ericsson P, Håkanson R, Rehfeld JF, Norlén P (2010). Gastrin release: antrum microdialysis reveals a complex neural control. *Regulatory Peptides*.

[94] Glavin GB (1989). Activity of selective dopamine DA1 and DA2 agonists and antagonists on experimental gastric lesions and gastric acid secretion. *Journal of Pharmacology and Experimental Therapeutics*.

[95] Chandranath SI, Bastaki SMA, D’Souza A, Adem A, Singh J (2011). Attenuation of stress-induced gastric lesions by lansoprazole, PD-136450 and ranitidine in rats. *Molecular and Cellular Biochemistry*.

[96] Brzozowski T, Konturek PC, Chlopicki S (2008). Therapeutic potential of 1-methylnicotinamide against acute gastric lesions induced by stress: role of endogenous prostacyclin and sensory nerves. *Journal of Pharmacology and Experimental Therapeutics*.

[97] Yigiter M, Albayrak Y, Polat B, Suleyman B, Salman AB, Suleyman H (2010). Influence of adrenal hormones in the occurrence and prevention of stress ulcers. *Journal of Pediatric Surgery*.

[98] Choung RS, Talley NJ (2008). Epidemiology and clinical presentation of stress-related peptic damage and chronic peptic ulcer. *Current Molecular Medicine*.

[99] Fink G (2011). Stress controversies: post-traumatic stress disorder, hippocampal volume, gastroduodenal ulceration. *Journal of Neuroendocrinology*.

[100] Schubert ML (2009). Gastric exocrine and endocrine secretion. *Current Opinion in Gastroenterology*.

[101] Strang RR (1965). The association of gastro-duodenal ulceration and Parkinson’s disease. *The Medical Journal of Australia*.

[102] Szabo S (1979). Dopamine disorder in duodenal ulceration. *The Lancet*.

[103] Chung ES, Chung YC, Bok E (2010). Fluoxetine prevents LPS-induced degeneration of nigral dopaminergic neurons by inhibiting microglia-mediated oxidative stress. *Brain Research*.

[104] Kumar A, Prakash A, Pahwa D (2011). Galantamine potentiates the protective effect of rofecoxib and caffeic acid against intrahippocampal Kainic acid-induced cognitive dysfunction in rat. *Brain Research Bulletin*.

[105] Roghani M, Niknam A, Jalali-Nadoushan MR, Kiasalari Z, Khalili M, Baluchnejadmojarad T (2010). Oral pelargonidin exerts dose-dependent neuroprotection in 6-hydroxydopamine rat model of hemi-parkinsonism. *Brain Research Bulletin*.

[106] Glavin GB (1991). Dopamine and gastroprotection. The brain-gut axis. *Digestive Diseases and Sciences*.

[107] Mayer EA (2000). The neurobiology of stress and gastrointestinal disease. *Gut*.

[108] Gray SJ, Benson JA, Reifenstein RW (1951). Chronic stress and peptic ulcer. I. Effect of corticotropin (ACTH) and cortisone on gastric secretion. *The Journal of the American Medical Association*.

[109] Willems JL, Buylaert WA, Lefebvre RA, Bogaert MG (1985). Neuronal dopamine receptors on autonomic ganglia and sympathetic nerves and dopamine receptors in the gastrointestinal system. *Pharmacological Reviews*.

[110] Glavin GB (1992). Dopamine: a stress modulator in the brain and gut. *General Pharmacology*.

[111] Xing LP, Balaban C, Seaton J, Washington J, Kauffman G (1991). Mesolimbic dopamine mediates gastric mucosal protection by central neurotensin. *American Journal of Physiology*.

[112] Henke PG (1979). Limbic lesions and the energizing, aversive, and inhibitory effects of non-reward in rats. *Canadian Journal of Psychology*.

[113] Hoogerwerf W, Pasricha PJ, Brunton LL, Lazo JS, Parker KL (2006). Pharmacotherapy of gastric acidity, peptic ulcers, and gastroesophageal reflux disease. *The Pharmacological Basis of Therapeutics*.

[114] Valle JD, Fauci AS, Braunwald E, Kasper DL (2008). Peptic ulcer disease and related disorders. *Harrison’s Principles of Internal Medicine*.

